# Intimacy versus Isolation: A Qualitative Study of Sexual Practices among Sexually Active HIV-Infected Patients in HIV Care in Brazil, Thailand, and Zambia

**DOI:** 10.1371/journal.pone.0120957

**Published:** 2015-03-20

**Authors:** Elizabeth F. Closson, Matthew J. Mimiaga, Susan G. Sherman, Arunrat Tangmunkongvorakul, Ruth K. Friedman, Mohammed Limbada, Ayana T. Moore, Kriengkrai Srithanaviboonchai, Carla A. Alves, Sarah Roberts, Catherine E. Oldenburg, Vanessa Elharrar, Kenneth H. Mayer, Steven A. Safren, for the HPTN063 study team

**Affiliations:** 1 The Fenway Institute, Fenway Health, Boston, Massachusetts, United States of America; 2 Harvard Medical School/Massachusetts General Hospital, Boston, Massachusetts, United States of America; 3 Harvard School of Public Health, Boston, Massachusetts, United States of America; 4 Johns Hopkins School of Public Health, Baltimore, Maryland, United States of America; 5 Research Institute for Health Sciences, Chiang Mai University, Chiang Mai, Thailand; 6 Instituto de Pesquisa Clinica Evandro Chagas, Fundação Oswaldo Cruz (Fiocruz), Rio de Janeiro, Brazil; 7 Centre for Infectious Disease Research in Zambia, Lusaka, Zambia; 8 FHI360, Durham, North Carolina, United States of America; 9 Faculty of Medicine, Chiang Mai University, Chiang Mai, Thailand; 10 National Institute of Allergy and Infectious Disease, Bethesda, Maryland, United States of America; 11 Harvard Medical School/Beth Israel Deaconess Medical Center, Boston, Massachusetts, United States of America; Catholic University of Sacred Heart of Rome, ITALY

## Abstract

The success of global treatment as prevention (TasP) efforts for individuals living with HIV/AIDS (PLWHA) is dependent on successful implementation, and therefore the appropriate contribution of social and behavioral science to these efforts. Understanding the psychosocial context of condomless sex among PLWHA could shed light on effective points of intervention. HPTN 063 was an observational mixed-methods study of sexually active, in-care PLWHA in Thailand, Zambia, and Brazil as a foundation for integrating secondary HIV prevention into HIV treatment. From 2010–2012, 80 qualitative interviews were conducted with PLWHA receiving HIV care and reported recent sexual risk. Thirty men who have sex with women (MSW) and 30 women who have sex with men (WSM) participated in equal numbers across the sites. Thailand and Brazil also enrolled 20 biologically-born men who have sex with men (MSM). Part of the interview focused on the impact of HIV on sexual practices and relationships. Interviews were recorded, transcribed, translated into English and examined using qualitative descriptive analysis. The mean age was 25 (SD = 3.2). There were numerous similarities in experiences and attitudes between MSM, MSW and WSM across the three settings. Participants had a high degree of HIV transmission risk awareness and practiced some protective sexual behaviors such as reduced sexual activity, increased use of condoms, and external ejaculation. Themes related to risk behavior can be categorized according to struggles for intimacy and fears of isolation, including: fear of infecting a sex partner, guilt about sex, sexual communication difficulty, HIV-stigma, and worry about sexual partnerships. Emphasizing sexual health, intimacy and protective practices as components of nonjudgmental sex-positive secondary HIV prevention interventions is recommended. For in-care PLWHA, this approach has the potential to support TasP. The overlap of themes across groups and countries indicates that similar intervention content may be effective for a range of settings.

## Introduction

Despite an overall decline in new HIV infections globally, identification of strategies to reduce HIV transmission among people living with HIV/AIDS (PLWHA) remains a major public health priority [1]. Antiretroviral treatment-as-prevention (TasP) interventions have offered promising results in serodiscordant heterosexual couples as evidenced by the dramatic results of the HPTN052 study [2]. However, not all patients in HIV treatment attain or sustain a suppressed viral load [3–5]. Comprehensive secondary HIV prevention interventions will need to address behavioral factors such as assuring treatment adherence, controlling sexually transmitted co-infections, and promoting condom use for prevention of primary HIV transmission or superinfection [6]. Secondary HIV prevention efforts will be the most powerful when administered as a combination of biomedical and behavioral approaches interventions [7–9].

While previous studies have shown a reduction in condomless sex immediately following HIV diagnosis [10–12], reports have also suggested that a substantial proportion of HIV-infected individuals do continue to engage in condomless anal and vaginal sex after their HIV diagnosis [13–16]. Brazil, Thailand, and Zambia, all have HIV epidemics that are primarily driven by sexual transmission, despite experiencing different HIV epidemics. General population prevalence estimates between the countries vary widely—0.6% in Brazil, 0.8% in Thailand, and 10.4% in Zambia [1]. There is an emerging body of research exploring the sexual behaviors of PLWHA in each of the three settings. Factors such as substance use, gender norms around sexual negotiation, instability in sexual relationships, being on antiretroviral therapy (ART), and the number and characteristics of sex partners have all found to be associated with rates of condomless sex among PLWHA in Brazil, Thailand, and/or Zambia [17–23]. However, most of our current knowledge comes from quantitative studies. One notable exception (among a handful of others) is a qualitative study conducted by Kerrigan et al. (2006) among PLWHA in Rio de Janeiro, Brazil [24]. Findings showed that sexual risk-taking was framed for many PLWHA as a pathway for social validation and was linked to intense feelings of shame about HIV, but also in a more general sense. Moreover, participants in the study expressed considerable fear and anxiety about disclosing their HIV status to sex partners. As this and other studies like it suggest, in-depth qualitative research that seeks to contextualize the sexual lives and relationship dynamics of PLWHA in these settings has the potential to enrich our understanding of these important issues.

The present study considers the perceived impact of HIV on the sexual practices and relationships of in-care HIV-infected men who have sex with men (MSM) and heterosexual men (MSW) and women (WSM) in Thailand, Brazil, and Zambia who engaged in high-risk sexual practices. To gain information about how to extend secondary prevention efforts to these settings, and add contextually relevant behavioral components to TasP efforts, this qualitative study was conducted as part of a mixed methods multi-site preparatory research initiative across demographically and geographically distinct populations of PLWHA engaged in HIV care. Analogous to the work of psychologist Eric Erikson, we frame our findings in relation to the stage of human development that is defined by the need to establish intimate relationships. The presence of HIV introduces distinct challenges to intimacy and thus has important implications for the psychosocial development of PLWHA.

## Methods

### Ethics Statement

Study procedures were approved by the at Johns Hopkins School of Public Health Institutional Review Board (Thailand), the Instituto de Pesquisa Clinica Evandro Chagas IRB (Brazil), the Comissão Nacional de Ética em Pesquisa (Brazil), the University of Alabama at Birminham IRB (Zambia), the HIV Prevention Trials Network (HPTN) Protocol Review Committee, and the Division of Acquired Immunodeficiency Syndrome DAIDS Prevention Science Review Committee. All participants were explained the purpose of the study, the voluntary nature of participation, and its risks and benefits and provided written informed consent before the start of data collection. Thai and Zambian participants received a small monetary incentive for partaking in the qualitative interview. Those in Brazil received a food/travel voucher. Staff members underwent training in research ethics and qualitative interviewing skills. To preserve confidentiality, data were de-identified and stored in a secure location and securely transferred electronically to investigators involved in the analysis.

### Participants and Procedures

Semi-structured qualitative individual interviews were conducted between June 2010 and June 2012 with in-care PLWHA from Chiang Mai, Thailand, Rio de Janeiro, Brazil, and Lusaka, Zambia. A total of 80 participants were interviewed, including 30 MSW (10 per site), 30 WSM (10 per site), and 20 MSM (10 each at the Chiang Mai and Rio sites). Note that for study inclusion criteria, those in the MSM category were to be biologically born men who currently identify as having sex with men. Accordingly, five individuals who identified as transgender were included. This qualitative study was one component of HPTN063 which also had a longitudinal quantitative component (N = 751; analysis underway). Involvement in the qualitative study was voluntary. Recruitment for this qualitative study followed a selective sampling technique whereby, study staff approached participants in the parent study who they thought would be willing to share their experiences in a qualitative interview. Individuals were recruited for the study at hospitals and clinics where they received HIV care. They were approached by a member of the study team in the waiting room of the clinic or hospital. In Zambia, the setting was a local HIV clinic. In Brazil, the setting was a clinical research institute that also provides HIV care. In Chiang Mai, the site is part of a public university, and participants were recruited from several local HIV clinics. All sites had local community advisory boards (CABs) that helped shape the study procedures, questions, recruitment, and retention plans. Eligibility criteria for this study and the parent study were the same. Eligible individuals were over the age of 18, HIV infected, had attended at least two HIV-related medical appointments in a formal health care setting (clinic or hospital) in the prior nine months and reported sexual risk behavior (acquisition of a sexually transmitted infection, condomless vaginal or anal intercourse, difficulty negotiating condom use, or non-disclosure of HIV status to an HIV-uninfected sex partner or sex partner of unknown HIV serostatus) within 12 months of enrollment. Table 1 provides information on the socio-demographics and sexual practices reported by participants in the qualitative sub-study. The interviews lasted for approximately one hour and were conducted by trained research staff in a private space. They were audio recorded, reviewed for identifying information and transcribed verbatim. Transcripts were translated into English by a bilingual staff person and independently assessed for accuracy and consistency.

**Table 1:** Sociodemographic, behavioral, and health characteristics of participants (N = 80).

	Mean (SD)
**Age**	25 (3.2)
**Number of years diagnosed with HIV**	4.3 (3.4)
**Number of sex partners within the past 3 months**	4.1 (15.94)
	N *(%)*
**Gender**	
Males	45 (56.2)
Female	30 (37.5)
Transgender woman (enrolled as MSM)	5 (6.3)
**Sexual orientation identity**	
Heterosexual or “Straight”	60 (75)
Homosexual or Gay	20 (25)
**Marital status**	
Married	35 (43.7)
Never married	32 (40)
Divorced/separated	6 (7.5)
Widowed	7 (8.8)
**Education**	
Completed all or some of primary school	14 (17.5)
Completed all or some of secondary school	37 (46.3)
Completed technical training	9 (11.2)
Completed all or some of a university degree	20 (25)
**Unemployed**	13 (16.3)
**Took antiretroviral therapy in the last 3 months**	73 (91.3)
**Undetectable viral load**	43 (53.8)
**Condomless sex with an HIV-uninfected or unknown status sex partner within the past 3 months**	80 (100)

### Data Collection

Participants were given a brief interviewer-administered demographic questionnaire. This was followed by a semi-structured qualitative interview that was conducted in participants’ preferred local language and guided by a pre-established set of open-ended questions and optional scripted probes. Participants were asked questions on the following domains: (1) individual barriers and facilitators to safe sex (e.g., What has helped you engage in safer sexual practices in the past?); (2) sexual communication (e.g., What has it been like when you have you disclosed your HIV status to any sexual partners?); (3) HIV-related stigma (e.g., What are your fears about living with HIV in your community?); (4) structural factors influencing sexual risk behaviors (e.g., What kinds of pressures do you feel from your friends or people in your community about sex?); (5) Preferences for content and format of a future intervention to reduce sexual transmission risk. The present analysis focused on the perceived impact of HIV on participants’ sexual behavior and relationships with sex partners. With regard to this topic, participants were asked: How has HIV affected your sex life, if at all? Probes included: (1) Tell me about the types of sexual behaviors that you engage in; and (2) How has your use of condoms changed after you were diagnosed, if at all? Interview guides were pilot tested with study staff and community advisory board members for site-specific cultural competency. All of the interview data collected for the study was considered in the analysis.

### Analytical Approach and Data Analysis

Data analysis was conducted by the U.S. investigators with close collaboration from a qualitative committee comprised of staff members from each study site. A descriptive qualitative approach was used to characterize and describe these data at their natural level [25, 26]. Initial descriptive codes were identified through open coding and data immersion of three transcripts per risk group (MSM, MSW, and WSM) across all three study locations, for a total of nine transcripts. Codes were used to capture and organize significant statements (e.g., units of meaning consisting of words, phrases, and sentences). A list of codes was compiled, each with a definition and example quotation [27]. Two coders double coded four additional transcripts guided by the draft coding schema. The results of which were compared for consistency in text segmentation and code application. After establishing 95% agreement, the remaining transcripts were divided and coded by the two coders using Atlas *ti* qualitative analysis software (version 6.1). On-going discussions between the coders and the qualitative committee members helped to conceptualize emergent themes. The qualitative committee was comprised of investigators and the staff members who conducted the interviews.

## Results

By comparing the data across the sites and risk groups, we identified commonalities and variations in participants’ sexual practices, attitudes towards sex, and sexual relationships. The results of these qualitative data are presented according to three themes: (1) protective behaviors; (2) barriers to engaging in sexual safety; and (3) HIV stigma and sexual partnerships.

### Protective Behaviors

Participants described many changes in their sexual behavior as a consequence of becoming infected with HIV, including: reduction in sexual activity; increased focus on condom use; and engagement in lower-risk sexual activities. While several shared experiences across the three risk groups (MSW, WSM, and MSM) and sites (Brazil, Thailand, and Zambia) emerged, the data also reflected some notable distinctions.

#### Reduced sexual activity

The most salient protective theme that emerged was a reduction in sexual activity with both primary and casual sex partners after being diagnosed with HIV. Specifically, participants noted that they had sex more frequently prior to their diagnosis. Across sites and risk groups, reduced sexual activity was primarily attributed to a lack of interest in sex and diminished sexual pleasure. Some participants explained that their worries about transmitting HIV to their sex partner(s) interfered with their ability to enjoy sex and reduced their chances of having intimate sexual relationships.

In my case personally, I have become lesser interactive in terms of sexual life because I think I know the consequences, for example I meet a person, an opposite sex and I know that I would infect this person, I think that it would be very unfair for me to do that. In that case, I think…. normally…I don’t get interested in that [sex]. -*53 year-old Zambian MSW*

Relatedly, across all three sites and especially among WSM, diminished sexual pleasure was linked to feelings of guilt about sex.

Sex isn’t as good as it was, there’s no will to have it as I used to have it. It is always uneasy: ‘I’m doing something that it isn’t correct.’ But because in their mind everything is fine, so everything is perfect. But it’s too hard, it changes very much, our mind isn’t the same as it was before knowing we have the disease. -*28 year-old Brazilian WSM*

Some WSM, particularly in Thailand and Zambia, noted that after their diagnosis they acquired a distinct dislike for men and sex.

… From the time I tested positive and I hated men it has taken me quite a good number of years to talk kindly of my late husband, but because of being a Christian we learn about forgiveness and all these things; I just hated men, I hated sex, even this man that [I] am staying with, I thank God for him because he understands me. I don’t in fact enjoy [sex], at times I just do it to… satisfy him. -*50 year-old Zambia WSM*

A few MSM and MSW participants in each of the three settings noted that they experienced a decrease in sexual desire when they started taking antiretroviral therapy (ART).

I don’t know if it’s due to the antiretroviral drug, if it’s psychological, but I lost my senses to be horny… After I found out [my] HIV [status]… I started to take the drugs [ART], I don’t feel pleasure anymore. -*39 year-old Brazilian MSW*

Across risk groups and settings, a handful of participants attributed a reduction in sexual activity or the number of sex partners to a change in personal focus or “life style” after being diagnosed with HIV.

I mean, there was a drop I don’t know how many percent. It was a huge drop. But at the same time it is something that frustrates me, it gives me time to think about my values. Because, since I don’t base myself in my capacity of having sexual partners, which gave me social status of having many women. Going somewhere with one, then some other place with another. Going to a friend’s house with one, then another. Now I can’t do it anymore. This gives me the opportunity to build my emotional stability on other concepts. It is bad, yes, on the physical side, more immediate, but to me, I’m talking about me. Everyone is different. -*47 year-old Brazilian MSW*

MSW and WSM also noted that they reduced their number of sex partners after learning about some of the risk reduction strategies for PLWHA. This was particularly the case in Zambia.

At least now my sexual life has reduced after I knew my HIV status, after the education which I have gone through, I understand that this is true so I have to reduce on my sexual activities. -*44 year-old Zambian WSM*

MSM, MSW and WSM from all of the sites described an initial period of abstinence directly after they were infected with HIV. Feelings of shock, fear of transmission to sex partners, and worry about the need to disclose their HIV status had put them off sex for a while. This was particularly common for MSM, MSW, and WSM in Thailand and Brazil.

It’s just a period of time that I had less sexual practice. “Less” means I was scared of having sex. There was a period of time where I was conflicted with my own feelings. For example, I met a guy and I wanted to go there. What should I say? The word “having sex” is intertwined with feelings and relationships. The relationship too. Why didn’t I have sex with this guy? As an HIV infected person. And I didn’t use a condom. Might make him get infected. -*31 year-old Thai MSM*

However, at all sites, some participants, mostly MSM and MSW, noted that after they had been living with HIV for a while, their sexual activity increased or even returned to what it had been pre-diagnosis.

My sex frequency fairly decreased when I first knew about my HIV status because I couldn’t make up my mind at that time. So, I didn’t want to have sex or anything like that. But after I got back on track physically and mentally, then I can live normal as I used to. I can somehow have sex, like… it’s quite normal. -*32 year-old Thai MSM*

#### Increased focus on condom use

Participants described changes in condom use once they became infected with HIV. While an inclusion criterion of the study was engagement in high-risk sexual practices a large number of PLWHA said that they made an exerted effort to use condoms consistently since being diagnosed with HIV. Many said that they now thought more about condoms or risk reduction strategies whenever they thought about sex.

A MSW from Brazil said that even when he didn’t use a condom, he was thinking about them.

The only difference is that…We always have that little corner of our mind where we keep thinking: “Damn man…” Even when I’m not using a condom I’ll keep that in mind: “Damn it, I was supposed to use it”. It happens. Sometimes we feel that way but these are things that you can control mentally. You see? You make an internal control and learn to deal with this sort of thing. -*47 year-old Brazilian MSW*

Regardless of whether or not they knew the status of their sex partner, a few MSM, MWS and WSM from all three countries said that they used condoms to avoid reinfecting themselves with a different strain of the HIV virus.

If the seroconcordant couples don’t use condoms, they may be infected by the drug-resistant strains since the strains of virus can be varied among HIV-infected individuals, no matter if the individuals are males or females. -*52 year-old Thai WSM*

#### Engagement in lower-risk sexual practices

Consistent condom use was difficult for many of the participants. When condoms were not used, MSM and MSW, particularly in Thailand and Brazil, said that they actively made choices about reducing risk to their sex partners by engaging in lower risk sexual practices. For example, participants reportedly avoided ejaculating inside a sex partner during anal, vaginal and oral sex when condoms were not used.

Only that we don’t go up to the end, for example, we don’t have the final pleasure. If there is penetration we avoid the pleasure. Right? When we’re almost at climax we stop, interrupt to do the things outside not letting to be inside us, nor… If I have oral sex [my partner doesn’t] swallow the sperm. -*53 year-old Brazilian MSM who identified as a transgender* woman

MSM, MSW and WSM said that they actively tried to avoid vaginal and/or anal sex and engaged in non-penetrative sex if a condom was not used. A Thai MSM described trying different types of “soft sex” when he did not have a condom:

It’s a way of having soft sex. Soft sex means a sex without insertion. But it still makes you cum. Jerk each other off. Oral sex for each other. Lick ass. Jerking off each other, fooling around in that area. Just no insertion. It helps reduce risk. But it’s ok. There is no risk for HIV, but not for STIs anyway. -*31 year-old Thai MSM*

Some Brazilian MSW and WSM perceived anal sex as riskier than vaginal sex. A MSW reported that he sometimes had condomless vaginal sex, but made an effort to refrain from having condomless anal sex.

I don’t have anal sex without a condom. It has happened that I was having [vaginal] sex, [and I] wanted to have anal sex. I tried to put the condom [on] only to have anal sex. I always had [vaginal sex] without condom. Because in their [partner’s] head they’ll only have… Even knowing that there is HIV infection, which is my case. In their head the disease won’t be transmitted through [vaginal] sex. -*37 year-old Brazilian MSW*

### Barriers to engaging in sexual safety

Most of the participants found consistent condom use to be challenging. One of the primary sources of difficulty was that casual and primary sex partners viewed condomless sex as ‘normal’.

I have problems convincing them to use a condom every time because they (sex partners) are used to a system of having sex without condoms through and through. -*33 year-old Zambian MSW*

According to several participants, in light of the social norms around condomless sex, discussions around condoms generally centered on reasons why the couple should use a condom, as opposed to why they should forego condom use. As a 36 year-old Brazilian WSM remarked, “The partner never wants to use it (a condom). They want a reason to use condom. But we don’t need to have one”. For quite a few, it was difficult to talk to sex partners about condom use without arousing suspicion—either of having HIV and/or being unfaithful.

Another thing is unfaithfulness. There are people that cannot be trusted and others hide their [HIV] status from their partners. So using condoms makes them feel as though their status would be known. When a partner talks about condoms they respond, “You think that I am sick because I want to use a condom? I do not want to or maybe you are sick yourself so we should use condoms?” They pretend as though they do not know their status. At times stigma is the cause. -*32 year-old Zambian MSW*

A good number of PLWHA said that they did not initiate condom use with their partners because they wanted to avoid conversations about it. Participants explained that their partners often had negative reactions when they tried to persuade them to use a condom and that these interactions were awkward or contentious.

I was afraid that he would know…He may suspect. It’s like…If I hand him a condom, he would ask, “Why do you need to use a condom, anything wrong with you?” This thought has occupied me and makes me worry about using condoms. -*48 year-old Thai MSM*

Persuading a partner to use a condom during these intimate situations was particularly challenging for WSM.

It’s difficult to convince men to use condoms in the moment of desire. If their desire is sparked, we can’t stop them [and say], “Wait! You must wear a condom,” something like that. We can never stop them. I’ve experienced that myself. -*34 year-old Thai WSM*

The expectation of the potentially negative reaction by a partner may have been a reason for why WSM avoided discussions around condom use altogether.

MSM, MSW, WSM described a number of other impediments to condom use with primary sex partners. Many had trouble using condoms with primary sex partners because of a perceived lack of intimacy during sex with a condom. This was mostly relevant for MSW.

So practicing safer sex is not there as then it will mean your woman is holding something or you will be holding something from the woman, so you have to give it all to her and herself to you so when you use safer sex it means there is a problem between the two of you. Therefore it means problems in the house, so just do it like that no safer sex. -*31 year-old Zambian MSW*

The participant suggests that condom use represents ‘problems’ in the relationship. This idea likely stems from the social norms on condomless sex. As we described above, participants said that partners often wanted a ‘reason’ to use a condom. Infidelity is perceived as one of the main explanations for the need to use a condom in primary sexual partnerships. In this context, condom use infers or becomes symbolic of domestic strife and a loss of intimacy with in the relationship.

Similarly, introducing condoms into relationships where the couple was accustomed to condomless sex presented a distinct challenge for many participants.

I think I have no tendency to do that [use condoms]. It’s okay for me. She doesn’t say anything about that. I don’t use condoms with her and we’re living together like other normal couples. I’ve never use condoms with my wife from the start. If we used condoms from the start, I would use them now. -*44 year-old Thai MSW*

### HIV stigma and sexual relationships

PLWHA at all sites discussed the effects of HIV on relationships with sex partners. HIV stigma was the most common thread in discussions about relationships with sex partners. A significant theme, especially among WSM, was the need to avoid primary intimate sexual relationships because of worry about how a past or potential partner would react after HIV status disclosure.

We talked by telephone and didn’t expose our relationship. And then I used to think that …if he came to me one day…. It was before I had [HIV] infection. We already had slept together and I didn’t get infected at that time. Since getting infected I hadn’t met him for several years. But there is a chance to meet him. So I think about the ways to avoid him. If he wants to come, I will say “I’m busy right now” I try to talk to him to not let him come to see me. -*50 year-old Thai WSM*

PLWHA from all sites not only feared rejection from potential intimate partners but also worried that people outside of the relationships (e.g., people in the community) would find out about their HIV status, leading to stigma-related isolation. As a WSM from Brazil who reportedly went for long periods of time without having sex said:

Well, for a long time it was standby because I was so scared even with me because I thought that no one would accept me. It is also a risk to tell someone at once and this person is from your neighborhood and may tell other people. And you become the talk of the town. -*45 year-old Brazilian WSM*

Among MSM, MSW and WSM across the three sites, avoiding stigma was one of the main motivations for seeking a primary sex partner who was also living with HIV. Many described the benefit of a seroconcordant sex partner in terms of “acceptance” and not having to worry about rejection or HIV disclosure.

There are so many people out there that are after her but she just can’t accept [them] because she is HIV positive and she doesn’t have the will and the courage to tell everyone that ‘I am positive’…So she might as well just not accept or bring in anyone in her life as a sexual partner. For me I knew her status and I was in a good position of satisfying her sexual desires. -*32 year-old Zambian MSW*

## Discussion

To our knowledge this multi-site study represents the first qualitative examination of sexual behavior and decision-making among demographically and culturally distinct communities of PLWHA in care. These data highlight a number of similarities in experiences and attitudes between MSM, MSW and WSM across the three settings. Table 2 provides a summary of the thematic similarities and differences between the risk groups and sites. Overall, participants revealed a significant degree of conscientiousness about HIV transmission risk and emphasized the importance of risk reduction efforts after being diagnosed with HIV. PLWHA practiced different strategies to reduce the risk of HIV transmission to a sex partner, including a decrease in the number of sex partners, as well as an increase in condom use, external ejaculation, and non-penetrative sexual activities. However, the emphasis placed on external ejaculation as an effective risk reduction method suggests that many were not fully aware of the transmission risk presented by, for example, pre-ejaculate. None of the participants discussed pre-exposure prophylaxis (PrEP), and this was not available to potential partners at the study sites. Similarities also emerged in that participants across all settings discussed sexual safety in terms of their own health. Several emphasized the importance of condoms with seroconcordant primary partners due to the potential for reinfection with different strains of HIV. Nevertheless, use of condoms or other safer sex strategies proved difficult for a variety of reasons.

**Table 2:** Similarities and differences in themes according to risk groups and setting.

Themes	MSW			WSM			MSM		
	Thailand	Brazil	Zambia	Thailand	Brazil	Zambia	Thailand	Brazil	Zambia
**Reduced sexual activity due to lack of lack of sexual pleasure/interest in sex**									
Worries about transmitting HIV to partner	**O**	**O**	**O**	**O**	**O**	**O**	**O**	**O**	**O**
Feelings of guilt about sex				O	O	O			
Dislike for sex and men				O		O			
Reduced sexual desire when taking ART	O	O	O				O	O	O
Change in personal focus or “life style”	**O**	**O**	**O**	**O**	**O**	**O**	**O**	**O**	**O**
Period of abstinence immediately after diagnosis	O	O		O	O		O	O	
Sexual activity resumed to pre-diagnosis levels after living with HIV for an extended period of time	O	O	O				O	O	O
**Increased focus on condom use after diagnosis**											
Used condoms to avoid reinfection of HIV	**O**	**O**	**O**	**O**	**O**	**O**	**O**	**O**	**O**
Engagement in lower-risk sexual practices	O	O					O	O	
Perceived anal sex as less risky than vaginal sex		O			O				
**Barriers to engaging in sexual safety**									
Sex partners viewed condomless sex as the norm	**O**	**O**	**O**	**O**	**O**	**O**	**O**	**O**	**O**
Discussions about condom use aroused suspicion	**O**	**O**	**O**	**O**	**O**	**O**	**O**	**O**	**O**
Wished to avoid discussions around condom use altogether	**O**	**O**	**O**	**O**	**O**	**O**	**O**	**O**	**O**
Difficulty persuading partners to use a condom; feared negative response from partner				O	O	O			
Perceived lack of intimacy during sex with a condom	O	O	O						
Difficult to introduce condoms when couple was accustomed to condomless sex	**O**	**O**	**O**	**O**	**O**	**O**	**O**	**O**	**O**
**HIV stigma and sexual relationships**									
Avoided primary sexual partnerships out of fear that partner would react negatively when HIV status was disclosed				O	O	O			
Afraid that partner would disclose HIV status to others	**O**	**O**	**O**	**O**	**O**	**O**	**O**	**O**	**O**
Avoiding anticipated stigma was motivation for seeking a seroconcordant sexual relationship	**O**	**O**	**O**	**O**	**O**	**O**	**O**	**O**	**O**

O = Denotes similarities across risk groups and settings.

Many of the sexual practices and relationship considerations described by participants were related to a struggle for culturally embedded sexual intimacy and fear of isolation. Across the three sites, MSM, MSW and WSM described condom use as an obstacle to sexual intimacy between primary sex partners. WSM at all sites spoke about the unwillingness of primary partners to use condoms and the challenges of sexual communication in intimate situations. Consistent use of risk reduction techniques may also be particularly difficult for those who view them as impediments to sexual intimacy and satisfaction. These issues are likely connected to problems with introducing regular condom use into existing primary sexual partnerships. Fears of HIV-related stigma and consequent isolation colored conversations about relationships with sex partners and were not distinct to risk groups or site. In some cases, anxiety about intimacy resulted in participants shunning primary sexual relationships to limit the potential for rejection/isolation and avoid disclosure of HIV status. Also within the context of isolation, a central theme to emerge was reduced sexual activity after becoming infected with HIV. This was largely prompted by a decline in sexual desire that was related to worry about HIV transmission to a sex partner and guilt about sexual enjoyment. Although an underlying level of risk awareness motivates engagement in safer sex, excessive anxiety around sex may be connected to difficulties with sexual intimacy and is thought to exacerbate psychological distress for PLWHA [28]. Adverse mental health outcomes are known to reduce the effects of TasP efforts via suboptimal ART adherence and inconsistent condom use [12, 29–31].

This is not the first study to consider the sexual behaviors of PLWHA in each of the three countries. Studies among PLWHA in Brazil, Thailand, and Zambia have documented factors associated with patterns of condom use [17–24]. The present study builds upon some of these findings but also moves beyond them to highlight the thematic salience of intimacy and isolation in the sexual narratives of in-care PLWHA from these settings, and the similarities across various sites. Prior research has shown that HIV-infected women in the U.S. experience guilt about sex and worry about transmitting to sex partners [32]. In the U.S. and U.K., studies of PLWHA in long term relationships have emphasized the tension between intimacy and HIV prevention but have primarily focused on reproductive decision-making [33–35]. To date, however, there is limited data exploring these topics in developing world contexts. In a recent review article of the psychosocial factors that affect PLWHA across different settings, social isolation was identified as a theme in three studies [36]. However, two of these articles examine isolation in the context of non-disclosure to family, peers, and community [37, 38], and the other article discusses social support in the context of ART adherence [39]. By contrast, our study focuses on sexual activity and explores the themes of isolation, intimacy, stigma, and disclosure within the context of sexual decision-making. These qualitative data seek to contextualize the impact of HIV on sexual activity by looking at participants’ experiences, attitudes, and feelings around safer sex strategies, sexual communication, and the dynamics of sexual relationships.

This study has limitations. Firstly, to facilitate comparisons across sites, the interviews were semi-structured using qualitative interview guides. This type of interview format may have inhibited interviewers from probing for emergent themes that were not otherwise addressed in the guide. Great thought was given to develop interview guides that could meaningfully examine similar experiences in a diverse set of contexts. The guides were sufficiently uniform to allow for a comparison across the risk groups (MSM, MSW and WSM) and sites (Brazil, Thailand and Zambia) but had enough flexibility to encourage a natural flow of conversation. Second, there may have been slight variations in the interview guides based on language. To enhance data collection uniformity, interview trainings were conducted by the same investigators across the three sits. Additionally, English-language versions of the transcripts were used in the analysis. Although translations were verified for accuracy and completeness, there may have been some loss of information in the transcription process. Notably though, the analysis was conducted with significant input from a qualitative committee comprised of investigators and the staff members who conducted the interviewers who clarified context and meaning of quotations as necessary. All participants engaged in high-risk sexual practices and were enrolled in HIV medical care. This sample may not necessarily be representative of all PLWHA in the settings. Additionally, study staff selected in-care PLWHA for enrollment who they thought would be willing to share their experiences with the interviewer. Individuals who were shy and/or had difficulties talking about their sexual lives may represent a more vulnerable population. Finally, the study was not intended to attend to all of the socio-structural domains that undoubtedly influence sexual risk taking for MSM, MSW, WSM PLWHA in these countries.

Despite these limitations, these data offer a unique opportunity to deepen our understanding of the shared experiences of sexually active PLWHA with respect to barriers and facilitators of safer sexual practices across three distinct regions of the world. Behavioral interventions to reduce sexual risk for PLWHA, even for those who have achieved viral suppression, are critically important to maximizing and sustaining the effects of the success of TasP [8, 9]. However, secondary prevention efforts in international settings are limited and effective behavioral programming in diverse settings requires careful preparedness work. Our findings have the potential to inform the development of future context-specific behavioral components to support existing TasP efforts. Provider-led behavioral programs should acknowledge and build off of existing HIV transmission risk awareness and sense of personal responsibility for risk reduction that patients already have. Strength-based approaches have been shown not only to support provider-patient relationships but improve outcomes along the HIV care continuum [42]. Further, the thematic overlap in participants’ experiences and attitudes suggests that similar content could be suitable for care-based secondary HIV prevention interventions among MSM, MSW and WSM across a diverse array of settings. While adapting programs to the local socio-structural context is critical, grounding future intervention efforts in similar foundational content across international settings may better allow us to meet the challenges of expanded secondary prevention responses.

One possible explanatory model of our findings is Erik Erikson’s theory on the stages of psychosocial development [40]. According to Erikson, “Intimacy vs. Isolation” is one of a series of personal crises that individuals negotiate throughout their lives. Adults in this stage (usually aged 19–40) begin to share themselves more intimately with others with the goal of establishing meaningful relationships and a sense of commitment and care. Conversely, avoiding intimacy leads to isolation and a fear of self-disclosure, which can have negative lasting effects on future personal development. As participants’ mean age was 25, the majority was experiencing this Ericsonian stage while also living with HIV. Our findings suggest that HIV posed significant barriers to intimacy for MSM, MSW and WSM in all of the settings, such as worry of infecting sex partners, guilt about sexual enjoyment, HIV-related stigma, and struggles with sexual communication with intimate primary sex partners. Such manifestations are known to threaten sexual risk reduction efforts and engagement in HIV care. PLWHA may therefore benefit from sex-positive programs that underscore sexual health and intimacy as a critical component of secondary HIV prevention. Interventions designed to help PLWHA reduce sexual anxiety and guilt, engage in healthy sexual relationships and enhance sexual self-image may improve risk reduction self-efficacy and mental health over the long-term. Such an approach could augment the efforts of provider-led interventions focused on condom use, sex in the context of suppressed viral load, and optimum utilization of services in the HIV care cascade [41]. For those in a stable partnership, a couples-based approach may be well suited to address sexual partner intimacy in the context of secondary prevention. Couples-based interventions emphasizes the mutual responsibility for health of both partners and the role of the couple’s relationship in behavior change through the promotion of sexual communication and negation skills [42]. There are a growing number of studies demonstrating the advantages of the couples-based modality, yet further research is needed to identify strategies for integrating such an approach into the HIV care setting. Furthermore, this type of programme would likely be augmented by structural interventions focused on the gender-based power imbalances that influence sexual decision-making [43]. While Erikson’s theory may be a particularly relevant lens through which to view our findings, it should be underscored that there are myriad other orientations through which to frame them. For example, a sociological model may understand the observed struggles for intimacy within the context of seeking to achieve social normative status as it relates to cultural meanings of what it is to be connected or intimate. While the field of HIV prevention is fluid, qualitative studies that explore the sexual lives and practices of individuals living with HIV will be necessary to stay abreast of how to best promote interventions across the continuum of HIV care.
